# Adjunct Therapy with Ipragliflozin Exerts Limited Effects on Kidney Protection in Type 1 Diabetes: A Retrospective Study Conducted at 25 Centers in Japan (IPRA-CKD)

**DOI:** 10.3390/biomedicines13061287

**Published:** 2025-05-23

**Authors:** Yuta Nakamura, Ichiro Horie, Hiroshi Yano, Hiroshi Nomoto, Tomoyasu Fukui, Yoshihiko Yuyama, Tomoyuki Kawamura, Mariko Ueda, Akane Yamamoto, Yushi Hirota, Yoshiki Kusunoki, Kenro Nishida, Dan Sekiguchi, Yasutaka Maeda, Masae Minami, Ayako Nagayama, Shimpei Iwata, Hitomi Minagawa, Shinya Furukawa, Teruki Miyake, Hiroaki Ueno, Rei Chinen, Yoshiro Nakayama, Hiroaki Masuzaki, Yasutaka Miyachi, Yosuke Okada, Mitsuhiro Okamoto, Kaoru Ono, Ken-ichi Tanaka, Akira Kurozumi, Takenori Sakai, Hironori Yamasaki, Jun-ichi Yasui, Ayako Ito, Atsushi Kawakami, Norio Abiru

**Affiliations:** 1 Department of Endocrinology and Metabolism, Nagasaki University Hospital, Nagasaki 852-8501, Japan; y.nakamura@nagasaki-u.ac.jp (Y.N.); abirun@nagasaki-u.ac.jp (N.A.); 2 Clinical Research Center, Nagasaki University Hospital, Nagasaki 852-8501, Japan; 3 Department of Rheumatology, Endocrinology and Nephrology, Faculty of Medicine and Graduate School of Medicine, Hokkaido University, Sapporo 060-8638, Japan; 4 Division of Endocrinology, Metabolism, and Rheumatology, Department of Internal Medicine, Asahikawa Medical University, Asahikawa 078-8510, Japan; 5 Division of Diabetes, Metabolism, and Endocrinology, Department of Medicine, Showa University School of Medicine, Shinagawa 142-8555, Japan; 6 Seino Medical Clinic, Koriyama 963-8851, Japan; 7 Division of Pediatrics, Osaka Metropolitan University Graduate School, Osaka 545-8585, Japan; 8 Abeno Medical Clinic, Osaka 545-0052, Japan; 9 Division of Diabetes and Endocrinology, Department of Internal Medicine, Kobe University Graduate School of Medicine, Kobe 650-0017, Japan; 10 Department of Diabetes, Endocrinology and Clinical Immunology, School of Medicine, Hyogo Medical University, Nishinomiya 663-8501, Japan; 11 Division of Diabetes and Endocrinology, Kumamoto Central Hospital, Kumamoto 862-0965, Japan; 12 Minami Diabetes Clinical Research Center, Clinic Masae Minami, Fukuoka 815-0071, Japan; 13 Division of Endocrinology and Metabolism, Department of Internal Medicine, Kurume University School of Medicine, Kurume 830-0011, Japan; 14 Division of Endocrinology and Metabolism, Yame General Hospital, Yame 834-0034, Japan; 15 Division of Metabolism and Endocrinology, Faculty of Medicine, Saga University, Saga 849-8501, Japan; 16 Health Services Center, Ehime University, Bunkyo, Matsuyama 790-8577, Japan; 17 Department of Gastroenterology and Metabology, Ehime University Graduate School of Medicine, Toon 791-0295, Japan; 18 Furukawa Clinic, Matsuyama 790-0943, Japan; 19 Division of Hematology, Diabetes, and Endocrinology, Department of Internal Medicine, Faculty of Medicine, University of Miyazaki, Miyazaki 889-1692, Japan; 20 Division of Endocrinology, Diabetes and Metabolism, Hematology, Rheumatology, Graduate School of Medicine, University of the Ryukyus, Nishihara 903-0215, Japan; 21 Department of Medicine and Bioregulatory Science, Graduate School of Medical Sciences, Kyushu University, Fukuoka 812-8582, Japan; 22 First Department of Internal Medicine, School of Medicine, University of Occupational and Environmental Health, Kitakyushu 807-8555, Japan; 23 Department of Endocrinology, Metabolism, Rheumatology and Nephrology, Faculty of Medicine, Oita University, Oita 879-5593, Japan; 24 Department of Metabolic Medicine, Faculty of Life Sciences, Kumamoto University, Kumamoto 860-8556, Japan; 25 Department of Rheumatology and Diabetes, Wakamatsu Hospital of the University of Occupational and Environmental Health, Kitakyushu 808-0024, Japan; 26 Department of Internal Medicine, Yahatahama City General Hospital, Yahatahama 796-8502, Japan; 27 Department of Diabetes and Endocrinology, Sasebo General Medical Center, Sasebo 857-8511, Japan; 28 Department of Endocrinology and Metabolism, NHO Nagasaki Medical Center, Omura 856-0835, Japan; 29 Center of Diabetes and Endocrinology, Sasebo Central Hospital, Sasebo 857-1165, Japan; 30 Midori Clinic, Nagasaki 852-8034, Japan

**Keywords:** ipragliflozin, type 1 diabetes, SGLT2 inhibitor, chronic kidney disease, renal outcome

## Abstract

**Background/Objectives**: While sodium–glucose cotransporter 2 (SGLT2) inhibitors have demonstrated additional non-glycemic benefits for renal protection in individuals with type 2 diabetes, less evidence is available for those with type 1 diabetes (T1D). To determine whether the adjunctive use of the SGLT2 inhibitor ipragliflozin confers kidney protection in individuals with T1D, we retrospectively analyzed data from a real-world cohort examined at 25 centers in Japan. **Methods**: We enrolled 359 subjects aged 20–74 years with T1D (IPRA group: 159 ipragliflozin users; control [CTRL] group: 200 non-users). The primary outcome was changes in the estimated glomerular filtration rate (eGFR) from baseline to 24 months after the initiation of ipragliflozin. The secondary outcomes were all other changes, including the urinary albumin–creatinine ratio (UACR) and urinary protein–creatinine ratio (UPCR). **Results**: The IPRA group’s eGFR decline slopes were 0.79 mL/min/1.73 m^2^/year milder than the CTRL group’s after propensity score matching, but this difference was not significant. The subjects complicated by chronic kidney disease (CKD) defined as UACR ≥ 30 mg/g and/or UPCR ≥ 0.5 g/g and/or eGFR < 60 mL/min/1.73 m^2^ showed changes in UPCR (g/g) from baseline to 24 months that were significantly lower in the IPRA group (−0.27 ± 1.63) versus the CTRL group (0.18 ± 0.36) (*p* = 0.016). No significant increase in adverse events (including severe hypoglycemia and hospitalization due to ketosis/ketoacidosis or cardiovascular diseases) was observed in the IPRA group. **Conclusions**: Adjunctive treatment with ipragliflozin exerted potential renal benefits by decreasing proteinuria in T1D subjects with CKD. Further investigations are required to determine whether its additional benefits exceed the increased risk of ketoacidosis.

## 1. Introduction

Sodium–glucose cotransporter (SGLT) 2 inhibitors were originally developed to lower glucose levels for the treatment of individuals with type 2 diabetes (T2D) in an insulin-independent manner by inhibiting glucose reabsorption from the proximal renal tubules. Several large-scale randomized-controlled trials (RCTs) of SGLT2 inhibitors have demonstrated substantial benefits in cardiovascular and renal outcomes among subjects with T2D [1], and SGLT2 inhibitors have recently shown benefits in renal and heart failure even in individuals without diabetes [2,3,4].

An adjunctive treatment with SGLT2 inhibitors is considered a simple and effective option to fulfill unmet needs and complement insulin therapy in individuals with type 1 diabetes (T1D). Global clinical T1D trials have been conducted to examine additional indications for the use of SGLT2 inhibitors, including canagliflozin [5], dapagliflozin [6,7,8], empagliflozin [9], and sotagliflozin (a dual SGLT1/2 inhibitor) [10,11,12], for individuals with T1D. Phase 3 controlled clinical trials with two SGLT2 inhibitors, ipragliflozin [13] and dapagliflozin [14], among subjects with T1D have also been conducted in Japan. Meta-analyses of the RCTs on T1D have revealed that SGLT2 inhibitors cause significant reductions in glycated hemoglobin (HbA1c) levels, glycemic variability [15,16,17], insulin dose, body weight, and systolic blood pressure (SBP), without increasing the risk of hypoglycemia [18,19,20].

Although the adjunctive use of SGLT2 inhibitors has shown these benefits in T1D, the above-cited RCTs and meta-analyses have revealed a significantly increased risk of diabetic ketoacidosis (DKA) as a serious adverse event of these drugs [6,7,8,9,10,11,12,18,19,20]. The U.S. Food and Drug Administration (FDA) has never approved the use of any SGLT2 inhibitors for individuals with T1D. In Europe, the European Medicines Agency (EMA) and the National Institute for Health and Care Excellence (NICE) approved dapagliflozin and sotagliflozin for limited use in individuals with T1D with a body mass index (BMI) > 27 kg/m^2^; however, recently, the EMA and the NICE withdrew the T1D indication for these drugs [21].

In Japan, ipragliflozin and dapagliflozin were officially authorized as adjuncts to insulin therapy for adults with T1D in December 2018 and March 2019, respectively. These two SGLT2 inhibitors can now be used for individuals with T1D in Japan regardless of their BMI.

Guidelines from multiple professional societies declare that the cardiovascular and renal benefits provided by SGLT2 inhibitors have been established in individuals with T2D [22,23,24]. T1D and T2D may share metabolic, inflammatory, and hemodynamic mechanisms, and we speculated that treatment with an SGLT2 inhibitor may provide additional non-glycemic benefits to individuals with T1D as they have in those with T2D. However, none of the RCTs on SGLT2 inhibitors as a treatment for T1D were designed to evaluate cardiovascular/renal benefits as a primary outcome. In addition, the study durations of these RCTs (with a maximum of 52 weeks) might have been too short to evaluate the additional non-glycemic benefits of SGLT2 inhibitors in T1D [5,6,7,8,9,10,11,12,13,14]. Moreover, individuals with comorbidities such as chronic kidney disease (CKD), cardiovascular disease (CVD), and heart failure were excluded from these trials [5,6,7,8,9,10,11,12,13,14]. As there are currently few regions/countries other than Japan where SGLT2 inhibitors are officially approved for use in T1D populations, the real-world evidence concerning the additional benefits of SGLT2 inhibitors in T1D is limited.

We conducted the present multicenter retrospective cohort study to investigate the real-world outcomes of kidney function by evaluating changes in the estimated glomerular filtration rate (eGFR), albuminuria, and proteinuria before and after the adjunctive use of ipragliflozin in individuals with T1D.

## 2. Materials and Methods

### 2.1. Subjects

The inclusion criteria for the candidate subjects treated with ipragliflozin (the IPRA group) were as follows: (i) age ≥ 20 and <75 years; (ii) duration of T1D ≥ 5 years (60 months); (iii) initiation of ipragliflozin treatment ≥ 24 months ago; (iv) existing medical records ≥ 12 months before and ≥24 months after the initiation of ipragliflozin were available; and (v) the candidate was an outpatient.

The inclusion criteria for candidate subjects who were not treated with an SGLT2 inhibitor (the control [CTRL] group) were as follows: (i) age ≥ 20 and <75 years; (ii) duration of T1D ≥ 5 years (60 months); (iii) the candidate had not been administered any SGLT2 inhibitor for ≥5 years at enrollment; (iv) existing medical records ≥ 5 years before enrollment were available; and (v) the candidate was an outpatient.

Individuals were excluded if they met any of the following criteria: (i) a history of treatment for a malignancy (e.g., surgery, radiation, or chemotherapy) within 5 years at enrollment; (ii) a history of pregnancy or breast feeding within 5 years at enrollment; or (iii) alcohol consumption ≥ 20 g/day within 5 years at enrollment. We did not exclude individuals with T1D even if they had other diabetic complications (such as neuropathy or retinopathy) or other associated diseases.

### 2.2. Study Design

This was a retrospective cohort study carried out at 25 centers of the IPRA-CKD study group in Japan from June 2022 to July 2023. It was registered with the University Hospital Medical Information Network Clinical Trials Registry (UMIN-CTR, no. 000047441).

We aimed to enroll as many subjects with T1D into the IPRA group as possible and to enroll more subjects into the CTRL group due to the possibility of reductions in their numbers after propensity score matching.

The “index date” was defined as the date that a prescription of ipragliflozin was initially made for the members of the IPRA group. In this group, data obtained every 6 months during a 36-month period (from 12 months before to 24 months after the index date) were collected from the patients’ medical records. In the CTRL group, data obtained every 6 months during the 5-year (60-month) period before enrollment were collected, and the index date of this group was determined after collecting the last 5 years of data, as described in the Data Collection and Handling section below. All data collected from medical records were provided with a ±3-month variance from the determined day.

We collected the data regarding the participants’ clinical characteristics; medication regimen, including insulin and SGLT2 inhibitors; and laboratory data, including HbA1c, blood urea nitrogen (BUN), creatinine, eGFR, alanine aminotransferase (ALT), urinary ketone, urinary albumin-to-creatinine ratio (UACR), and urinary protein-to-creatine ratio (UPCR). All reported adverse events were registered, including severe hypoglycemia requiring any assistance from others, hospitalization due to ketosis/ketoacidosis, the development of a cardiovascular event, hospitalization due to heart failure and/or a cardiovascular event and/or kidney disease, and the initiation of hemodialysis during the participant’s data collection period. If a subject discontinued ipragliflozin within 24 months after initiation, the reasons for its discontinuation were collected from the medical records. The collected data were managed using the Research Electronic Data Capture system.

### 2.3. Data Collection and Handling During the COVID-19 Pandemic

In clinical practice in Japan, ipragliflozin has been generally prescribed for individuals with T1D since 2019, following its approval for treating T1D on 21 December 2018. The observational period for some members of the IPRA group overlaps with the coronavirus disease 2019 (COVID-19) pandemic. The government’s restrictions on people’s regular activities might have impacted the glycemic control and kidney function of the study subjects, and to minimize the bias from this impact, we adjusted the data period used for the CTRL group to match the data period used for the IPRA group. The index date for the CTRL group members was determined using the following five steps: (i) All subjects registered in the IPRA group were arranged in order of their index date and divided into four groups (quarters) according to this order. (ii) We named the periods divided into four parts based on the index date Periods 1, 2, 3, and 4, from earliest to latest. (iii) The index dates of the CTRL subjects were randomly assigned to one of Periods 1–4. (iv) If two or more data points were available during the period assigned to the index date, the date of the data that was closest to the period’s median was used as the subject’s index date. (v) The data obtained during the 36 months were used for the analyses, from 12 months before to 24 months after the assigned index date of the CTRL subjects.

### 2.4. Primary/Secondary Outcomes and Safety Endpoints

The primary outcome of this study was changes in eGFR values from baseline (the index date) to 24 months after the index date. The secondary outcomes were as follows: a >50% reduction in the eGFR; end-stage kidney disease (ESKD) defined as an eGFR < 30 mL/min/1.73 m^2^ and/or a requirement for hemodialysis; changes in the UACR, UPCR, HbA1c, body weight, and insulin requirement dose from baseline (the index date) to 24 months after the index date; early changes in the eGFR from baseline to 6 months after the index date; and chronic changes in the eGFR from 6 to 24 months after the index date.

This study’s safety endpoints were the prevalence of severe hypoglycemia requiring any assistance from others, hospitalization due to ketosis/ketoacidosis, and hospitalization due to CVD, including heart failure.

### 2.5. Statistical Analyses

The results are presented as the mean ± standard deviation (SD) or the median (interquartile range [IQR]) for the continuous values and the *n*-value (%) for the categorical variables. Wilcoxon’s rank sum test and the χ^2^ test were performed for the continuous and categorical variables, respectively. The differences between the post-index-date eGFR changes for the IPRA and CTRL groups were evaluated using Wilcoxon’s rank sum test. Probability (*p*)-values < 0.05 (two-tailed) were accepted as significant. The variables used in the development of the propensity scores included age, sex, HbA1c, eGFR, total insulin dose, tobacco smoking history, and use of angiotensin-converting-enzyme inhibitors (ACEIs)/angiotensin II receptor blockers (ARBs) at the index date.

The subjects in the CTRL group were matched with those in the IPRA group at a 1:1 ratio based on their propensity scores. We excluded subjects who discontinued ipragliflozin within 24 months after its initiation from the IPRA group before the propensity matching was performed. All analyses were performed with R ver. 4.3.2.

### 2.6. Ethical Considerations

This study was approved by the ethical committees of Nagasaki University Hospital (no. 22062204) and all of the study group’s participating centers. We conducted the present study in accordance with the Helsinki Declaration of 1964 and its later amendments. Written informed consent or consent through the opt-out recruitment method was obtained from the participants, depending on the regulations used by each center participating in the IPRA-CKD study.

## 3. Results

A total of 360 adults with T1D were recruited. However, 1 man withdrew his informed consent before enrollment, so a final total of 359 subjects (228 women and 131 men) were included in the study analyses. Of these, 159 subjects were treated with ipragliflozin (the IPRA group), and the other 200 were not treated with an SGLT2 inhibitor (the CTRL group). Table 1 summarizes the baseline characteristics of both groups. The IPRA group showed significantly higher BMI values (25.6 ± 3.6 vs. 23.0 ± 3.6 kg/m^2^, *p* < 0.001), diastolic blood pressure (DBP) (74 ± 12 vs. 71 ± 12 mmHg, *p* = 0.027), total insulin dose (47.3 ± 23.8 vs. 38.5 ± 17.1 units/day, *p* < 0.001), HbA1c (8.3 ± 1.1 vs. 7.7 ± 1.1%, *p* < 0.001), and proteinuria than the CTRL group. The frequencies of existing CKD, dyslipidemia, and statin use at baseline were significantly higher in the IPRA group than in the CTRL group.

Seventeen subjects in the IPRA group (~10%) discontinued ipragliflozin within 24 months after its initiation. The reasons for discontinuation are listed in Supplemental Table S1. There were no significant differences in baseline characteristics between those who continued ipragliflozin and those who discontinued ipragliflozin (Supplemental Table S2). We excluded these 17 subjects from the propensity score matching as their ipragliflozin treatment period was <24 months. After propensity score matching, the cohort included 65 subjects in the IPRA group and 65 subjects in the CTRL group. All baseline characteristics were well matched, with no significant between-group differences (Table 1).

### 3.1. Primary Outcome

**Figure 1:**
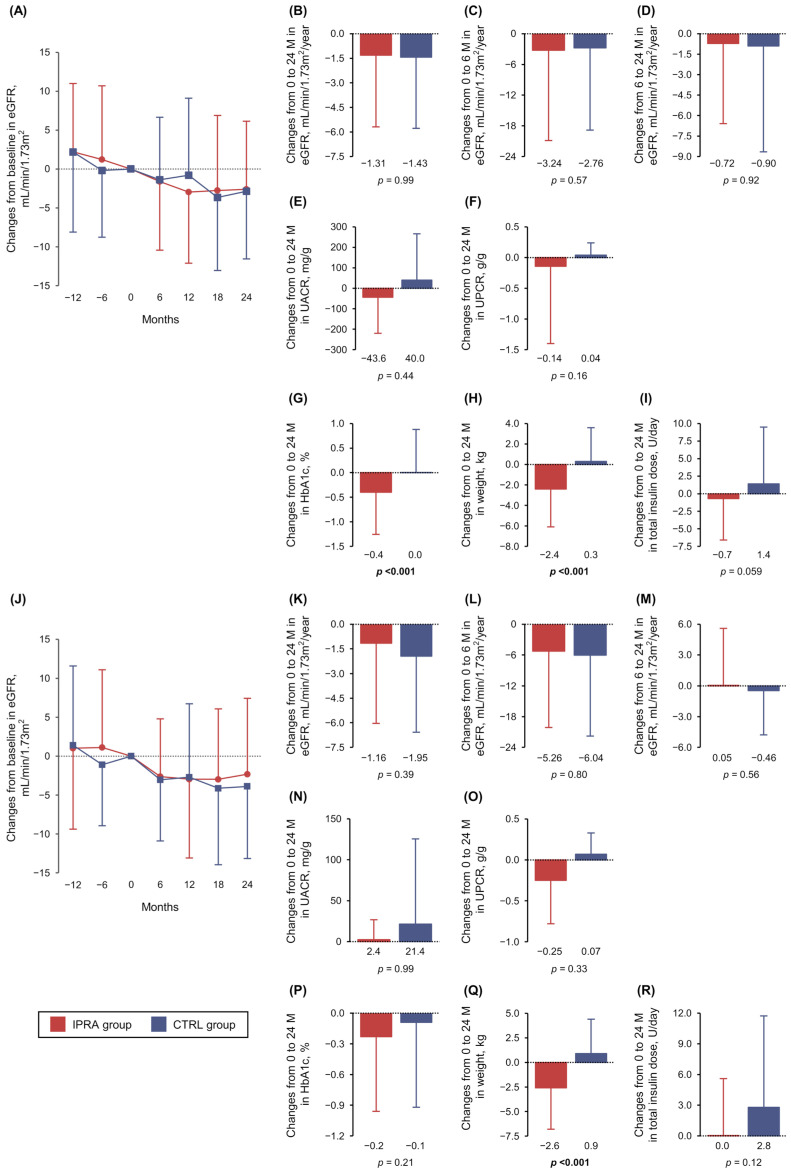
Subjects prior to (**A**–**I**) and after propensity score matching (**J**–**R**) in the IPRA group (red) and the CTRL group (blue). All line graphs and bar graphs indicate the means ± SDs. The mean changes in the eGFR over the period before and after the index date were calculated using Wilcoxon’s rank sum test (**A**,**J**) but were not significantly different. The annual rates of eGFR changes from baseline (the index date) to 24 months, those from baseline to 6 months, and those from 6 to 24 months after the index date are shown in (**B**–**D**,**K**–**M**). Changes in the UACR, UPCR, HbA1c, body weight, and total daily insulin dosage from baseline to 24 months after the index date are shown in (**E**–**I**,**N**–**R**). eGFR: estimated glomerular filtration rate; HbA1c: glycated hemoglobin; UACR: urinary albumin-to-creatinine ratio; UPCR: urinary protein-to-creatine ratio.

Figure 1A shows the changes in the eGFR over the 36 months before and after the index date in the IPRA and CTRL groups. The mean annual rates of eGFR change (mL/min/1.73 m^2^/year) from baseline to 24 months after the index date in the IPRA group (*n* = 159) and the CTRL group (*n* = 200) were −1.31 ± 4.38 and −1.43 ± 4.35, respectively, with no significant difference between the groups (*p* = 0.99) (Figure 1B).

After the propensity score matching was performed, the eGFR decline slope after the index date in the IPRA group tended to be milder than that of the CTRL group (Figure 1J). The mean annual rates of eGFR change from baseline to 24 months after the index date (mL/min/1.73 m^2^/year) in the IPRA group (*n* = 65) and the CTRL group (*n* = 65) were −1.16 ± 4.88 and −1.95 ± 4.63, respectively (Figure 1K). The eGFR slope reductions in the IPRA group were 0.79 mL/min/1.73 m^2^/year lower than those in the CTRL group, but the difference between the groups was not significant (*p* = 0.39).

In a post hoc mixed-effects model analysis of the IPRA group excluding the period during the first 6 months after the start of ipragliflozin, the eGFR slope during the 12 months before the index date was −3.00 mL/min/1.73 m^2^ per year, while the eGFR slope from 6 to 24 months after the index date was −1.31 mL/min/1.73 m^2^ per year. The decline in eGFR of the IPRA group tended to slow after initiating the ipragliflozin treatment (*p* = 0.090).

### 3.2. Secondary Outcomes

Between the baseline (the index date) and 24 months after the index date, no subjects in either the IPRA or CTRL group experienced a ≥50% decline in the eGFR, developed new-onset ESKD, or initiated hemodialysis. In addition, the changes in the eGFR from baseline to 6 months after the index date and the changes from 6 to 24 months after the index date were not significantly different between the IPRA and CTRL groups prior to (Figure 1C,D) or after the propensity score matching was performed (Figure 1L,M).

There were also no significant between-group differences in the changes in the UACR, UPCR, or insulin requirement dose from baseline to 24 months after the index date, evaluated before (Figure 1E,F) and after the propensity score matching (Figure 1N,O). However, the IPRA group’s HbA1c levels (%) and body weight (kg) decreased significantly compared with the CTRL group’s over the 24 months (−0.4 ± 0.9 vs. 0.0 ± 0.9, *p* < 0.001 in Figure 1G, and −2.4 ± 3.7 vs. 0.3 ± 3.3, *p* < 0.001 in Figure 1H), evaluated prior to the propensity score matching.

After the propensity score matching, no significant between-group difference was observed in the change in HbA1c levels (Figure 1P), but the significant difference in body weight (kg) remained after matching (−2.6 ± 4.2 for the IPRA group vs. 0.9 ± 3.5 for the CTRL group; *p* < 0.001) (Figure 1Q). The changes in the total insulin dosage from baseline to 24 months were not significantly different between the groups either before or after the matching (Figure 1I,R).

### 3.3. Post Hoc Analyses

**Figure 2:**
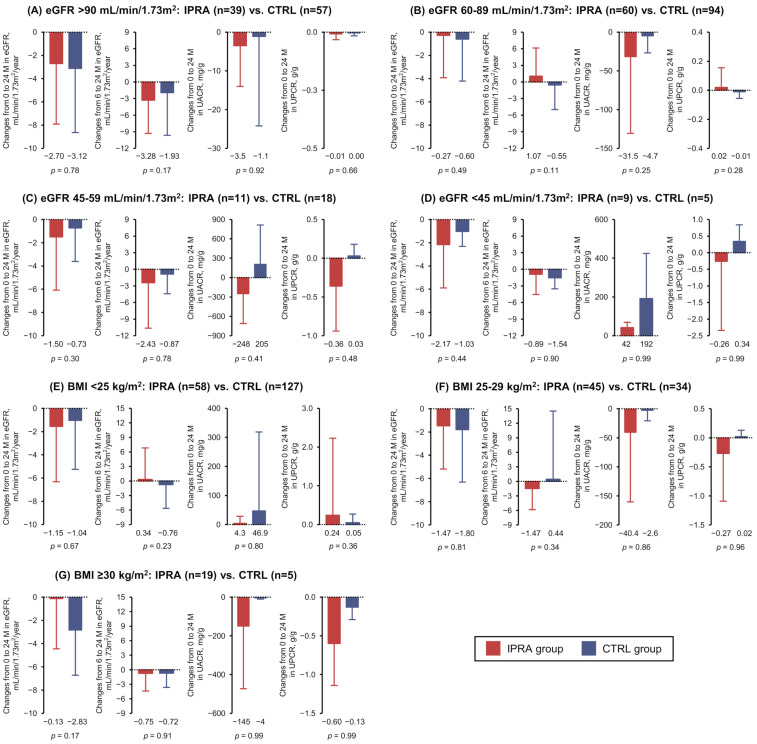
The annual rates of eGFR change from baseline to 24 months and those from 6 to 24 months after the index date and the changes in the UACR (mg/g) and UPCR (g/g) over the 24 months were determined in the subgroups based on baseline eGFR values (mL/min/1.73 m^2^) ≥ 90 (**A**), 60–89 (**B**), 45–59 (**C**), or <45 (**D**) and baseline BMI (kg/m^2^) <25 (**E**), 25–29 (**F**), or ≥30 (**G**) at the index date between the IPRA (red bars) and CTRL (blue bars) groups. All graphs indicate the means ± SDs. BMI: body mass index; eGFR: estimated glomerular filtration rate; UACR: urinary albumin-to-creatinine ratio; UPCR: urinary protein-to-creatine ratio.

We conducted subgroup analyses between the IPRA and CTRL groups, based on baseline eGFR values ≥ 90, 60–89, 45–59, and <45 mL/min/1.73 m^2^ and on baseline BMI values < 25 (normal weight), 25–29 (overweight), and ≥30 kg/m^2^ (obesity). The annual rates of eGFR decline from the baseline to 24 months after the index date were not significantly different between the IPRA and CTRL groups, irrespective of the subgroups (Figure 2). The annual rates of eGFR decline from baseline to 6 months and those from 6 to 24 months were also not significantly different between the IPRA and CTRL groups (Figure 2). The changes in the UACR and UPCR over 24 months between the IPRA and CTRL groups were not significantly different, irrespective of the subgroups (Figure 2).

**Figure 3:**
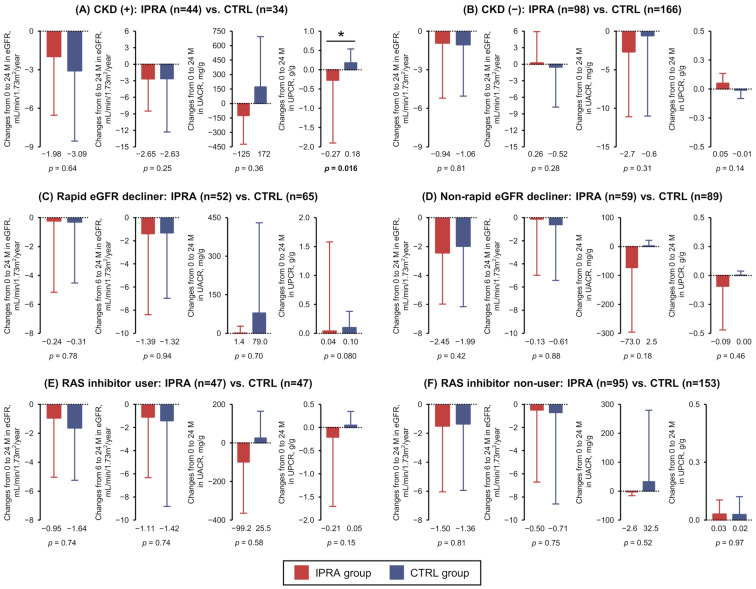
The annual rates of change in the eGFR from baseline to 24 months and those from 6 to 24 months after the index date and changes in the UACR (mg/g) and UPCR (g/g) over the 24 months were determined in the subgroups based on the presence/absence of CKD (**A**,**B**), subgroups with or without a rapid decline in the eGFR (>3 mL/min/1.73 m^2^/year) over the 12-month period before the index date (**C**,**D**), and subgroups with or without RAS inhibitor use (**E**,**F**) in the IPRA (red bars) and CTRL (blue bars) groups. All graphs indicate the means ± SDs. * indicates a significant *p*-value between the IPRA and CTRL groups. CKD: chronic kidney disease; eGFR: estimated glomerular filtration rate; RAS: renin–angiotensin system; UACR: urinary albumin-to-creatinine ratio; UPCR: urinary protein-to-creatine ratio.

In this cohort, 78 (44 in the IPRA group and 34 in the CTRL group) of 80 subjects whose baseline (the index date) status was complicated by CKD (defined by UACR ≥ 30 mg/g and/or proteinuria, and/or UPCR ≥ 0.5 g/g and/or eGFR < 60 mL/min/1.73 m^2^) had complete data on kidney functions over the study period. The analysis of these 78 subjects with CKD demonstrated that the eGFR decline slopes in the total period (from baseline to 24 months) and the chronic phase (from 6 to 24 months) were comparable between the groups (Figure 3A). The changes in UACR (mg/g) from baseline to 24 months were not significantly different between the IPRA and CTRL groups (−125.3 ± 297.2 vs. 171.6 ± 521.8, *p* = 0.36), but the changes in UPCR (g/g) differed significantly (−0.27 ± 1.63 vs. 0.18 ± 0.36, *p* = 0.016) (Figure 3A). By contrast, there were no significant between-group differences in the eGFR slope, UACR, or UPCR after the index date in the subjects without CKD at baseline (Figure 3B).

Our analyses of the subjects divided into groups of “rapid eGFR decliners”, defined by an annual decline in the eGFR > 3.0 mL/min/1.73 m^2^ at the index date, and the “non-rapid eGFR decliners” (Figure 3C,D), and of the subjects divided into renin–angiotensin system (RAS) inhibitor users and non-users (Figure 3E,F), revealed no significant differences in the changes in the eGFR, UACR, or UPCR between the IPRA and CTRL groups.

### 3.4. Safety Endpoints

As shown in Table 2, the prevalence of severe hypoglycemia requiring any assistance from others during the study period (from baseline to 24 months after the index date) was 3.1% in the IPRA group and 7.0% in the CTRL group, with no significance (*p* = 0.153). The rates of hospitalization due to ketosis/ketoacidosis or due to CVD were 1.3% and 0.6% in the IPRA group and 2.0% and 0.5% in the CTRL group, respectively, but were not significantly different between the groups (*p* = 0.70 and 0.99, respectively). The comparable safety outcomes between the IPRA and CTRL groups were replicated even in the analysis of subjects matched by propensity scores.

## 4. Discussion

This retrospective study of Japanese individuals with T1D demonstrated that adjunctive therapy with ipragliflozin had a limited additional benefit in kidney outcomes. The patients treated with ipragliflozin showed a significant reduction in proteinuria compared with those not treated with this SGLT2 inhibitor only in a small subgroup complicated by CKD (defined as a UACR ≥ 30 mg/g and/or UPCR ≥ 0.5 g/g and/or eGFR < 60 mL/min/1.73 m^2^). However, ipragliflozin treatment showed no significant protection against eGFR decline over 24 months, even in the subgroup analyses.

We observed that the individuals treated with ipragliflozin had higher BMI values, insulin doses, and HbA1c levels than those not treated with any SGLT2 inhibitor. Most subjects treated with ipragliflozin did not present with CKD, exhibiting a mean baseline eGFR of 81.3 mL/min/1.73 m^2^, which was comparable to those not treated with an SGLT2 inhibitor. In clinical practice for individuals with T1D, the adjunctive use of SGLT2 inhibitors is mainly aimed at improving glycemic control by decreasing glucose variability, body weight, and insulin dosage. Consequently, a limited number of cases in the present cohort might have received ipragliflozin with the expectation of additional benefits, including kidney protection.

A recent meta-analysis of 11 RCTs that assessed the safety and efficacy endpoints of SGLT2 inhibitors in T1D demonstrated that the use of SGLT2 inhibitors resulted in a significant reduction in albuminuria (by 23.13%), and this effect was consistent across all of the SGLT2 inhibitors used [25]. In contrast, no significant positive impact of SGLT2 inhibitors on the decline in the eGFR was observed compared with placebo over the maximum 52-week study duration [25].

In Tandem1 and 2—phase 3 trials of sotagliflozin in individuals with T1D who had a mean baseline eGFR at almost 90 mL/min/1.73 m^2^—sotagliflozin treatment resulted in a significant initial reduction in the eGFR compared with a placebo by week 4 of treatment, but the eGFR slope after week 4 was comparable to those in the placebo group [26]. Eighty-five percent of the participants in these trials did not have albuminuria, but in the subgroup with baseline UACR values ≥ 30 mg/g, a significant reduction in the UACR was observed at week 24 and was sustained to week 52 in subjects treated with sotagliflozin compared with those treated with placebo [26].

Similar results were reported for phase 3 trials of T1D with empagliflozin (the EASE-2 and EASE-3 trials). Most participants did not have impaired renal function, showing 97 and 99 mL/min/1.73 m^2^ as the mean eGFR values at baseline, respectively, and 6.2 mg/g as the mean UACR in both trials. Empagliflozin treatment produced an acute and sustained (up to 52 weeks) reduction in the eGFR compared with a placebo. In the participants with a baseline UACR ≥ 30 mg/g, treatment with 10 mg and 25 mg of empagliflozin significantly reduced the UACR by 16% and 30%, respectively, but treatment with 2.5 mg of empagliflozin did not significantly reduce the UACR [27].

In a post hoc analysis of the participants showing baseline albuminuria with a UACR ≥ 30 mg/g evaluated in phase 3 trials of dapagliflozin for the treatment of T1D (the DEPICT-1 and DEPICT-2 trials), dapagliflozin treatment resulted in a significant dose-dependent reduction in the UACR over 52 weeks (−13.3% for the 5 mg dose and −31.1% for the 10 mg dose compared with a placebo). The mean eGFR in the trials’ participants was approximately 85 mL/min/1.73 m^2^ at baseline, and an initial decrease in the eGFR was observed with dapagliflozin treatment, which subsequently stabilized such that no significant difference compared with the placebo was observed over the study period [28].

It has been hypothesized that the kidney protection provided by SGLT2 inhibitors is caused mainly by a reduction in afferent arteriolar vasodilation via an improvement in inappropriate tubuloglomerular feedback [29]. It has also been reported that reductions in glomerular pressure with SGLT2 inhibitor treatment are reflected by an initial dip in the eGFR and reductions in albuminuria (proteinuria) in clinical trials regardless of the presence/absence of diabetes [2,3,4]. Stougaard et al. simulated CVD and ESKD risk in subjects with T1D receiving adjunctive therapy with SGLT2 inhibitors using Steno Type 1 Risk Engines, a predictive model of CVD development in T1D [30]. In that study, 5-year CVD risk and 5-year ESKD risk were significantly reduced in the subgroup with albuminuria. Therefore, the reduction in proteinuria in T1D subjects with CKD in our real-world study may well lead to cardio-renal-protective effects.

A statement by the U.S. National Kidney Foundation in collaboration with the FDA and EMA noted that a threshold UACR reduction of 21–27% or a threshold eGFR slope reduction of 0.5–1.0 mL/min/1.73 m^2^ per year provides a 97.5% positive predictive value for a nonzero benefit for kidney outcomes in cohort studies [31]. The treatment’s beneficial effect on albuminuria may be observed in a 6-month follow-up period only in patients with a baseline UACR > 30 mg/g, and the higher the baseline UACR, the greater the albuminuria changes [31]. Regarding the eGFR, a longer follow-up (≥2–3 years) may be required to estimate slopes that reliably predict a treatment effect; in addition, a negative acute effect with an initial dip that develops within 3 months can attenuate the statistical power advantage of the total slope [31].

In our cohort, the initial dip in the eGFR after administering ipragliflozin could not be evaluated because of a lack of data from the period within 3 months of the index date. After the propensity matching in this cohort, the eGFR slopes over 24 months in the IPRA group were 0.79 mL/min/1.73 m^2^/year lower than those observed in the CTRL group (Figure 1K). This difference did not reach statistical significance, perhaps due to insufficient statistical power, but it did exceed the threshold for eGFR slope reduction specified in the above-described statement and might be clinically meaningful. Considering these points, larger sample sizes and longer evaluation periods might be required to determine the precise additional effects of SGLT2 inhibitors on eGFR slope reduction in individuals with T1D.

Aside from the present study, a limited number of small-sample, short-period real-world data have been reported concerning the effect of SGLT2 inhibitors on renal function in T1D. Edwards et al. observed that SGLT2 inhibitors showed no apparent effect on the eGFR or albuminuria over a 12-month period in 39 subjects with T1D [32]. A retrospective cohort study conducted by Palanca et al. at two European centers evaluated the effect of SGLT2 inhibitors on the kidney functions of subjects with T1D (empagliflozin for 113 subjects, dapagliflozin for 66, and canagliflozin for 20) over a 12-month period, and the overall changes in the eGFR and UACR from baseline to 12 months were not significant. However, eGFR values increased significantly in subjects with a baseline eGFR < 90 mL/min/1.73 m^2^, and UACR values decreased significantly in subjects with a baseline UACR ≥ 15 mg/g over the study period [33].

Hironaka et al. recently described their multicenter retrospective cohort study of 76 Japanese subjects with T1D treated with dapagliflozin for 24 months [34]. Compared with our present cohort, their subjects had lower eGFR (74.0 mL/min/1.73 m^2^) and comparable UACR values (9.1 mg/g). They observed an initial dip and a subsequent lower decline in the eGFR and a lesser increase in the UACR among the dapagliflozin users compared with the non-users [34].

Considering these findings, including the present study, we suspect that SGLT2 inhibitors have additional effects on kidney protection even in individuals with T1D. However, it is too early to conclude whether such a renal-protective effect is achieved in all patients with any level of baseline eGFR and albuminuria (proteinuria) or whether the effect occurs independently of the type of SGLT2 inhibitor. Further RCTs enrolling subjects with CKD are necessary to determine whether the favorable effects of SGLT2 inhibitors on renal outcomes beyond the increased risk of ketoacidosis among individuals with T1D.

It has been hypothesized that mild but persistent hyperketonemia during treatment with an SGLT2 inhibitor results in significant risk reductions in both cardiovascular and renal outcomes in individuals with type 2 diabetes [35]. With the chronic use of SGLT2 inhibitors, increased β-hydroxybutyrate is effectively utilized in oxidized/damaged tubular cells by increasing ATP production [36] and suppressing the mechanistic target of rapamycin complex 1 signaling, which causes podocyte damage under diabetes [37]. The shift from glucose to fatty substrate utilization can improve the transduction of energy consumption into work efficiency at the mitochondrial metabolic level in proximal tubular cells, which may help to protect against renal events [37]. We previously revealed that ipragliflozin treatment increases the fasting β-hydroxybutyrate levels of individuals with T1D by approximately 2.5-fold [38]. Considering the elevated ketone levels, we speculate that treating T1D with SGLT2 inhibitors may provide additional benefits in cardiovascular and renal outcomes.

Our study has some limitations. (i) The baseline characteristics of the subjects in the IPRA and CTRL groups were significantly different, and quite a few participants had missing data; the number of cases was decreased after propensity score matching, which might have attenuated the statistical power. (ii) Most of the subjects did not have CKD, and this might not be suitable for evaluating the renal-protective effect of ipragliflozin. (iii) eGFR data within 3 months after the initiation of ipragliflozin treatment are necessary to confirm the typical initial eGFR dip after SGLT2i use, but these were missing. (iv) The 24-month observational period might be insufficient to make any conclusion regarding renal outcomes. (v) It is unknown whether our findings can be duplicated when studied in other ethnic groups or using other SGLT2 inhibitors.

## 5. Conclusions

This real-world study demonstrated that adjunctive treatment with ipragliflozin might have beneficial effects on kidney outcomes through decreasing proteinuria in subjects with T1D whose status is complicated by CKD. Further studies with more subjects and a longer observational period are necessary to evaluate the additional effects of ipragliflozin and other SGLT2 inhibitors on renal protection in T1D. Moreover, additional research is required to ascertain whether the adjunctive use of SGLT2 inhibitors provides significant benefits for kidney outcomes beyond the concern of an increased risk of ketoacidosis in individuals with T1D.

## Figures and Tables

**Table 1:** Baseline characteristics of the participants at the index date.

	IPRA Group	CTRL Group	*p*-Value
N	159	200	
Age, years	46.2 ± 12.7	48.1 ± 14.3	0.10
Male/female, *n*	58/101	73/127	0.99
Duration of diabetes, yrs	16.7 ± 10.0	16.1 ± 10.3	0.37
BMI, kg/m^2^	25.6 ± 3.6	23.0 ± 3.6	<0.001
SBP, mmHg	127 ± 16	124 ± 18	0.30
DBP, mmHg	74 ± 12	71 ± 12	0.027
Total insulin, unit/day	47.3 ± 23.8	38.5 ± 17.1	<0.001
HbA1c, %	8.3 ± 1.1	7.7 ± 1.1	<0.001
eGFR, mL/min 1.73 m^2^	81.3 ± 21.9	81.4 ± 20.9	0.98
UACR, mg/gCr	11.0 (5.0, 84.1)	11.7 (5.4, 23.0)	0.33
UPCR, g/gCr	0.54 (0.06, 1.13)	0.07 (0.04, 0.13)	0.027
Neuropathy—yes, *n* (%)	45 (28.3)	54 (27.0)	0.81
Retinopathy—yes, *n* (%)	54 (34.0)	52 (26.0)	0.11
CKD—yes, *n* (%)	46 (28.9)	34 (17.0)	0.008
CVD—yes, *n* (%)	9 (5.7)	8 (4.0)	0.47
Hypertension—yes, *n* (%)	63 (39.6)	73 (36.5)	0.59
Dyslipidemia—yes, *n* (%)	78 (49.1)	67 (33.5)	0.003
Smoking—yes, *n* (%)	53 (33.3)	61 (30.5)	0.57
Severe hypoglycemia—yes, *n* (%)	21 (13.2)	44 (22.0)	0.038
History of ketoacidosis—yes, *n* (%)	33 (20.8)	52 (26.0)	0.26
RAS inhibitor—yes, *n* (%)	51 (32.1)	47 (23.5)	0.075
Statin—yes, *n* (%)	59 (37.1)	43 (21.5)	0.001
After propensity score matching			
N	65	65	
Age, years	49.0 ± 12.7	48.4 ± 14.9	0.89
Male/female, *n*	23/42	23/42	0.99
Duration of diabetes, yrs	15.0 ± 9.4	16.9 ± 11.3	0.45
BMI, kg/m^2^	24.8 ± 3.2	24.8 ± 4.0	0.55
SBP, mmHg	129 ± 18	130 ± 16	0.41
DBP, mmHg	74 ± 11	73 ± 13	0.57
Total insulin, U/day	43.2 ± 21.3	42.2 ± 19.5	0.87
HbA1c, %	7.9 ± 1.0	8.0 ± 1.2	0.85
eGFR, mL/min 1.73 m^2^	81.2 ± 21.8	79.4 ± 22.9	0.58
UACR, mg/gCr	14.1 (5.1, 84.9)	12.8 (6.4, 21.1)	0.72
UPCR, g/gCr	0.13 (0.06, 0.97)	0.08 (0.05, 0.12)	0.41
Neuropathy—yes, *n* (%)	17 (26.2)	24 (36.9)	0.26
Retinopathy—yes, *n* (%)	23 (35.4)	22 (33.8)	0.99
CKD—yes, *n* (%)	18 (27.7)	13 (20.0)	0.41
CVD—yes, *n* (%)	4 (6.2)	1 (1.5)	0.37
Hypertension—yes, *n* (%)	23 (35.4)	34 (52.3)	0.08
Dyslipidemia—yes, *n* (%)	28 (43.1)	25 (38.5)	0.72
Smoking—yes, *n* (%)	21 (32.3)	21 (32.3)	0.99
Severe hypoglycemia—yes, *n* (%)	11 (16.9)	12 (18.5)	0.99
History of ketoacidosis—yes, *n* (%)	13 (20.0)	12 (18.5)	0.99
RAS inhibitor—yes, *n* (%)	21 (32.3)	21 (32.3)	0.99
Statin—yes, *n* (%)	25 (38.5)	17 (26.2)	0.19

Data are the mean ± standard deviation or median (interquartile range) for the continuous values and *n* (%) for the categorical variables. The *p*-values for the ipragliflozin (IPRA) group vs. the control (CTRL) group in all data were calculated using Fisher’s exact test or Wilcoxon’s rank sum test. Propensity scores were estimated controlling for age, sex, eGFR, total insulin, HbA1c, smoking history, and use of RAS inhibitors. BMI: body mass index; CKD: chronic kidney disease; CVD: cardiovascular disease; DBP: diastolic blood pressure; eGFR: estimated glomerular filtration rate; HbA1c: glycated hemoglobin; RAS: renin–angiotensin system; SBP: systolic blood pressure; UACR: urinary albumin-to-creatinine ratio; UPCR: urinary protein-to-creatine ratio.

**Table 2:** Adverse events during the study period.

	IPRA Group	CTRL Group	*p*-Value
N	159	200	
Severe hypoglycemia	5 (3.1)	14 (7.0)	0.15
Hospitalization due to ketosis/ketoacidosis	2 (1.3)	4 (2.0)	0.70
Hospitalization due to CVD	1 (0.6)	1 (0.5)	0.99
Urinary tract infections	4 (2.5)	1 (0.5)	0.18
Gastrointestinal symptoms	2 (1.3)	3 (1.5)	0.99
Dizziness/deafness	3 (1.9)	2 (1.0)	0.66
Dehydration	0 (0.0)	4 (2.0)	0.13
Upper respiratory tract infection	1 (0.6)	3 (1.5)	0.63
Oral infection	0 (0.0)	3 (1.5)	0.26
Weight loss	2 (1.3)	0 (0.0)	0.20
Skin rash	1 (0.6)	1 (0.5)	0.99
Liver dysfunction	1 (0.6)	1 (0.5)	0.99
Gout	1 (0.6)	1 (0.5)	0.99
After propensity score matching			
N	65	65	
Severe hypoglycemia	2 (3.1)	2 (3.1)	0.99
Hospitalization due to ketosis/ketoacidosis	0 (0.0)	2 (3.1)	0.50
Hospitalization due to CVD	0 (0.0)	0 (0.0)	NA
Urinary tract infections	3 (4.6)	1 (1.5)	0.62
Gastrointestinal symptoms	0 (0.0)	0 (0.0)	NA
Dizziness/deafness	2 (3.1)	0 (0.0)	0.50
Dehydration	0 (0.0)	2 (3.1)	0.50
Upper respiratory tract infection	1 (1.5)	0 (0.0)	0.99
Oral infection	0 (0.0)	2 (3.1)	0.50
Weight loss	1 (1.5)	0 (0.0)	0.99
Skin rash	1 (1.5)	1 (1.5)	0.99
Liver dysfunction	1 (1.5)	1 (1.5)	0.99
Gout	0 (0.0)	0 (0.0)	NA

Data are *n* (%). The *p*-values for the ipragliflozin (IPRA) group vs. the control (CTRL) group in all data were calculated using Fisher’s exact test. CVD, cardiovascular disease; NA, not applicable.

## Data Availability

The datasets generated and/or analyzed during this study are available from the corresponding author upon reasonable request.

## Supplementary Materials

The following supporting information can be downloaded at https://www.mdpi.com/article/10.3390/biomedicines13061287/s1: Table S1: Reasons for discontinuing ipragliflozin within 24 months post-initiation in the IPRA group (*n* = 159). Table S2: Baseline characteristics of the IPRA continuation group and IPRA discontinuation group at the index date.

## Abbreviations

The following abbreviations are used in this manuscript:

ACE	Angiotensin-converting-enzyme inhibitor
ARB	Angiotensin II receptor blocker
BMI	Body mass index
CKD	Chronic kidney disease
COVID-19	Coronavirus disease 2019
CVD	Cardiovascular disease
DKA	Diabetic ketoacidosis
eGFR	Estimated glomerular filtration rate
ESKD	End-stage kidney disease
HbA1c	Glycated hemoglobin
SGLT	Sodium–glucose cotransporter
T1D	Type 1 diabetes
T2D	Type 2 diabetes
UACR	Urinary albumin-to-creatinine ratio
UPCR	Urinary protein-to-creatine ratio
