# Supplementary material for: Adjunct Therapy with Ipragliflozin Exerts Limited Effects on Kidney Protection in Type 1 Diabetes: A Retrospective Study Conducted at 25 Centers in Japan (IPRA-CKD)
Source: Biomedicines. 2025 May 23;13(6):1287. doi: 10.3390/biomedicines13061287 (PMC12189841; doi:10.3390/biomedicines13061287)
Supplement: Supplementary file 1 [file biomedicines-13-01287-s001.zip › Supplemental Table S1 Nakamura et al.pdf]

Supplemental Table S1. Reasons for discontinuing ipragliflozin within 24 months post-initiation in the IPRA group (*n*=159)

Ketosis/Ketoacidosis	2 (1.3)
Hypoglycemia	2 (1.3)
Genital or urinary tract infections	3 (1.9)
Weight loss	2 (1.3)
Introduction of insulin pump	1 (0.6)
Development of Graves' disease	1 (0.6)
Thyroid dysfunction	1 (0.6)
Ipragliflozin unavailability	1 (0.6)
Participation in another clinical study	1 (0.6)
Withdrawal request	2 (1.3)
Unknown	1 (0.6)
Total	17 (10.7)

Data are *n* (%).
