# Supplementary material for: Adjunct Therapy with Ipragliflozin Exerts Limited Effects on Kidney Protection in Type 1 Diabetes: A Retrospective Study Conducted at 25 Centers in Japan (IPRA-CKD)
Source: Biomedicines. 2025 May 23;13(6):1287. doi: 10.3390/biomedicines13061287 (PMC12189841; doi:10.3390/biomedicines13061287)
Supplement: Supplementary file 1 [file biomedicines-13-01287-s001.zip › Supplemental Table S2 Nakamura et al.pdf]

Supplemental Table S2. Baseline characteristics of IPRA continuation group and IPRA discontinuation group at the index date

	**Continuation**	**Discontinuation**	***P*-value**
N	142	17	
Age, yrs	46.2 ± 12.7	46.2 ± 12.9	0.99
Male/female, *n*	53/89	5/12	0.60
Duration of diabetes, yrs	16.6 ± 10.0	17.5 ± 9.5	0.62
BMI, kg/m^2	25.8 ± 3.7	24.5 ± 2.9	0.20
SBP, mmHg	128 ± 16	121 ± 13	0.15
DBP, mmHg	74 ± 12	73 ± 11	0.82
Total insulin, unit/day	48.2 ± 24.5	39.8 ± 15.6	0.19
HbA1c, %	8.4 ± 1.2	8.2 ± 1.1	0.47
HbA1c, mmol/mol	68 ± 13	66 ± 12	0.47
eGFR, mL/min1.73m^2	80.8 ± 22.1	85.0 ± 20.1	0.62
UACR, mg/gCr	12.0 (4.9, 88.2)	8.4 (5.0, 17.6)	0.56
UPCR, g/gCr	0.54 (0.06, 1.07)	0.63 (0.06, 1.19)	0.91
Neuropathy—yes, *n* (%)	41 (28.9)	4 (23.5)	0.78
Retinopathy—yes, *n* (%)	47 (33.1)	7 (41.2)	0.59
CKD—yes, *n* (%)	44 (31.0)	2 (11.8)	0.16
CVD—yes, *n* (%)	9 (6.3)	0 (0.0)	0.60
Hypertension—yes, *n* (%)	56 (39.4)	7 (41.2)	0.99
Dyslipidemia—yes, *n* (%)	71 (50.0)	7 (41.2)	0.61
Smoking—yes, *n* (%)	46 (32.4)	7 (41.2)	0.59
Severe hypoglycemia—yes, *n* (%)	20 (14.1)	1 (5.9)	0.70
History of ketoacidosis—yes, *n* (%)	32 (22.5)	1 (5.9)	0.20
RAS inhibitor—yes, *n* (%)	47 (33.1)	4 (23.5)	0.59
Statin—yes, *n* (%)	54 (38.0)	5 (29.4)	0.60

Data are the mean ± standard deviation or median (interquartile range) for the continuous values and *n* (%) for the categorical variables. The *p*-values for the ipragliflozin continuation group vs. the ipragliflozin discontinuation group in all data were calculated using Fisher's exact test or Wilcoxon's rank sum test. BMI: body mass index; CKD: chronic kidney disease; CVD: cardiovascular disease; DBP: diastolic blood pressure; eGFR: estimated glomerular filtration rate; HbA1c: glycated hemoglobin; RAS: renin–angiotensin system; SBP: systolic blood pressure; UACR: urinary albumin-to-creatinine ratio; UPCR: urinary protein-to-creatine ratio.
